# Protein aerosol for intranasal nose to brain (N2B) delivery

**DOI:** 10.1186/1753-6561-9-S9-O11

**Published:** 2015-12-14

**Authors:** Martina Stützle, Stefan Carle, Lucas Engelhardt, Ulrich Simon, Annette Schafmeister, Chrystelle Mavoungou, Katharina Schindowski

**Affiliations:** 1 Institute of Applied Biotechnology, Biberach University of Applied Sciences, 88400Biberach, Germany; 2 Medical Faculty, Ulm University, 89081 Ulm, Germany; 3 Scientific Computing Centre Ulm, Ulm University, 89081 Ulm, Germany

## Background

For the treatment of metabolic and neurodegenerative diseases, drug delivery to the central nervous system (CNS) has gained considerable recent interest[1,2]. But many concepts for the delivery of therapeutic agents have failed by the natural blood-brain barrier (BBB). The N2B route for drugs is able to provide an adequate alternative of CNS drug delivery -bypassing the BBB[3,4]. N2B drug transport takes mainly place at the olfactory cleft, an area that is well hidden in the skull base [5]. In a previous study, we determined the characteristics of aerosols for optimal deposition for N2B delivery by a computational approach and validated those data with experimental deposition in a human nose model generated by rapid prototyping [manuscript in preparation]. With the determined criteria, different aerosol generators were evaluated for protein dispersion. Moreover, a surrogate method was developed that imitates the dispersion effect was used for formulation screening to maintain stability and function and to avoid shear stress during dispersion. The most promising protein formulations were tested in an in vitro exposition system for passage through a nasal epithelium in a transwell culture. This test system can be used to develop an N2B platform approach.

## Material and methods

The Carleton-civic standardized human nose model [6]was constructed from CT scans and displays the complex 3D geometry of the human nose and its olfactory cleft. The deposition of liquid airborne aerosols was simulated numerically by computational fluid and particle dynamics (CFPD). Those data were validated experimentally in a positive rapid prototyped human nose model. A surrogate agitation method, imitating the dispersion effect was applied for formulation screening within a Design of Experiment (DoE) approach using trehalose (0.5-6 % (w/v)), sorbitol (0.5-5 % (w/v)), arginine (0.5 - 5 % (w/v)), tween (0.005-0.05 % (v/v)) and cyclodextrin (0.35-3.5 % (v/v)). Protein stability was evaluated applying SE-HPLC and photometry to determine monomer content before and after dispersion with a vibrating mesh nebulizer (Aeroneb Pro, Aerogen 112 Inc., Galway, Ireland). Identified suitable formulations were exposed to a nasal epithelial cell line (RPMI 2650) growing on transwell inserts to investigate transport ratios. Therefore, various seeding densities and transwells were tested under submers and air-liquid interface (ALI) conditions. The confluence of a monolayer was investigated with confocal microscopy by staining adhesion and tight junction proteins and a valid transport model was established with FITC-dextran in the Vitrocell® Cloud System (Waldkirch, Germany). Finally, two formulations were further analyzed in detail to study molecular weight, concentration and formulation dependencies on transport ratios.

## Results and discussion

In the present study, we found that aerosol parameters had to be defined very precisely to enable deposition at the anatomically hidden olfactory cleft. Validation of CFPD simulation by the experimental nose model determined a particle size of 5-10 µm and a flow rate of 5-20 L/min as optimal conditions for improved deposition.

However, aerosol generation may cause shear stress that correlates with protein instability. Therefore, a vibrating mesh nebulizer was identified as suitable aerosol source that fulfills the requirements and specifications concerning particle size, flow rate, chemical and physical properties as well as material and cost. Nevertheless, dispersion is time and material consuming and hence, a surrogate method was established by agitation in a 96-well plate. An antibody Fab fragment was diluted in simple PBS formulations containing either cyclodextrin (0.35 and 3.5 % (w/v)), tween (0.05, 0.01, 0.005 % (v/v)) or arginine (2, 5 and 8 % (w/v)) and they were agitated at 900 rpm, 30 °C for 20 min. Turbidities after dispersion and agitation were measured and showed a significant correlation of R2 = 0.8688. Using the surrogate method, the formulation screen led to suitable formulations. The most promising formulation 1 (trehalose (1 % (w/v)), sorbitol (4% (w/v)), arginine (5% (w/v)), tween (0.028 %(v/v)) and cyclodextrin (0.35% (w/v))) revealed improved protein stability compared to the parental formulation (PBS) for different antibody formats as Fab, IgG A and IgG B after dispersion (Table 1).

**Table 1:** Formulation and stability: Fab, IgG A and IgG B were dispersed and analyzed via SE-HPLC comparing monomer content before and after dispersion (n = 3, ± SD).

	Formulation 1		PBS	
Constructs	undispersed	dispersed	undispersed	dispersed
Fab	103.21 ± 3.62	90.27 ± 6.87 (ns)	100.00 ± 0.53	77.86 ± 2.44 (**)
IgG A	103.17 ± 0.12	101.27 ± 2.79 (ns)	100.00 ± 0.28	86.92 ± 2.99 (**)
IgG B	92.72 ± 4.51	74.88 ± 5.15 (**)	100.00 ± 3.09	77.15 ± 4.12 (***)

For statistical analysis unpaired two-tailed t-Test was applied to compare undispersed with dispersed samples for each formulation (**p<0.01, ***p<0.001).

**Figure 1:**
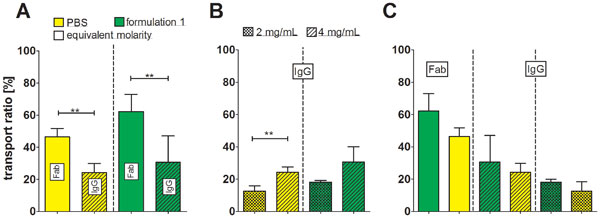
**Transport of protein aerosols in formulation 1 and PBS in a human nose epithelial cell model (n = 3, ± SD)**. A) transport ratios of different molecular weight sized proteins - Fab and IgG A. B) cells were exposed to two different IgG A concentrations. C) The influence of formulations on the transport ratio. For statistical analysis an unpaired two-tailed t-Test was applied (*p<0.05, **p<0.01, ***p<0.001).

To test the effects of different formulations on absorption on a nasal epithelium, we used a transwell system that is exposed to ALI. The system was characterized by FITC-dextran transport studies and confocal microscopy that confirmed tight junctions and a monolayer. IgG A and its Fab fragment was formulated and dispersed in the Vitrocell® Cloud system and transwells were transferred to the incubator for 4 h to study transport ratios. A correlation between molecular size and concentration was observed regarding transport ratios. Fab showed a significant improved diffusion compared to IgG A at equivalent molarities. Using two different IgG concentrations revealed a significant faster diffusion for the higher concentration (Figure 1).

## Conclusions

In conclusion, shear stress and dispersion effects had a strong impact on protein aggregation when generating micrometer-sized particles. Here, we present the establishment of a surrogate method imitating the dispersion effect and the subsequent formulation screen that led to improved protein stability after dispersion. Moreover, an *in vitro *exposition system was optimized to study transport ratios of different molecular weight sized proteins, concentrations and formulations. As aggregated proteins appear and cause a proinflammatory reaction, the nasal-epithelial cells will be co-cultivated with macrophages to study signs of immunogenicity after dispersion. These results will be verified in *in vivo*.

In summary, this test system is suitable to be used asN2B platform for protein aerosols. That includes particle size, deposition in experimental and *in silico *nose model, protein stability, *in vitro *and later on *in vivo *studies.
